# In situ recordings of large gelatinous spheres from NE Atlantic, and the first genetic confirmation of egg mass of *Illex coindetii* (Vérany, 1839) (Cephalopoda, Mollusca)

**DOI:** 10.1038/s41598-021-86164-8

**Published:** 2021-03-30

**Authors:** Halldis Ringvold, Morag Taite, A. Louise Allcock, Michael Vecchione, Michel Péan, Roberto Sandulli, Geir Johnsen, Arne Fjellheim, Snorre Bakke, Hanne Sannæs, Ann-Elin Wårøy Synnes, José Coronel, Martin Hansen, Peter G. Olejar, Geir Eliassen, Anita Eliassen, Karl Klungland

**Affiliations:** 1 Sea Snack Norway, Bergen, Norway; 2 School of Natural Sciences and Ryan Institute, National University of Ireland, Galway, Ireland; 3 NOAA/ Smithsonian Institution, Washington, USA; 4 DORIS, the naturalist website of the French Underwater Federation (FFESSM), Marseille, France; 5 CoNISMa, Parthenope University of Naples, Rome, Italy; 6 Norwegian University of Science and Technology, Trondheim, Norway; 7 NORCE research – Environment, Bergen, Norway; 8 Møreforsking AS, Ålesund, Norway; 9 Institute of Marine Research, Bergen, Norway; 10 Centre for Coastal Research, University of Agder, Kristiansand, Norway; 11 Mallorca, Palma, Spain; 12 Oceanfjord AS, Ålesund, Norway; 13 Ålesund Diving Club, Ålesund, Norway; 14 Arendal Underwater Club, Arendal, Norway; 15 Søgne Diving Club, Søgne, Norway

**Keywords:** Ecology, Genetics, Zoology, Ecology

## Abstract

In total, 90 gelatinous spheres, averaging one meter in diameter, have been recorded from ~ 1985 to 2019 from the NE Atlantic Ocean, including the Mediterranean Sea, using citizen science. More than 50% had a dark streak through center. They were recorded from the surface to ~ 60–70 m depth, mainly neutrally buoyant, in temperatures between 8 and 24°C. Lack of tissue samples has until now, prohibited confirmation of species. However, in 2019 scuba divers secured four tissue samples from the Norwegian coast. In the present study, DNA analysis using *COI* confirms species identity as the ommastrephid broadtail shortfin squid *Illex coindetii* (Vérany, 1839); these are the first confirmed records from the wild. Squid embryos at different stages were found in different egg masses: (1) recently fertilized eggs (stage ~ 3), (2) organogenesis (stages ~ 17–19 and ~ 23), and (3) developed embryo (stage ~ 30). Without tissue samples from each and every record for DNA corroboration we cannot be certain that all spherical egg masses are conspecific, or that the remaining 86 observed spheres belong to *Illex coindetii*. However, due to similar morphology and size of these spheres, relative to the four spheres with DNA analysis, we suspect that many of them were made by *I. coindetii*.

## Introduction

Rare, large (~ 1 m in diameter) gelatinous spheres from the NE Atlantic (Scandinavia and Mediterranean Sea), attributed to egg mass of ommastrephid squid (Oegopsida, Cephalopoda, Mollusca), have previously been reported^1^, but lack of tissue samples prevented molecular analysis and confirmation of species. Representatives of several squid families (e.g. Thysanoteuthidae Keferstein, 1866, Ommastrephidae Steenstrup, 1857 and possibly Lycoteuthidae Pfeffer, 1908) are known to produce large, neutrally buoyant structures, and egg masses of many squid families are unknown. Pelagic squid egg masses are rarely recorded, and studying in situ spheres is based on chance encounters. Such huge egg masses are thought to maintain their depth in the water column by floating on a pycnocline^2–9^.

Ommastrephids are the most abundant and widely distributed family of cephalopods, and are distributed throughout the world oceans from sub-Arctic seas to sub-Antarctic seas. They inhabit waters from the surface to depths of 2000 m (e.g. *Todarodes sagittatus* Steenstrup, 1880^10^), but are mainly recorded above 1000 m^11^. Three main ecological groups (life forms); (1) Slope-shelf group, (2) Nerito-oceanic group and (3) Oceanic group, are distinguished among ommastrephids, by characteristics of habitat, especially spawning habitat, which includes the degree of relationship with the bottom. Many species within *Illex*, *Todarodes*, *Todaropsis* and *Nototodarus* genera belongs within the slope-shelf group where the main habitats are the waters of the continental slope and shelf^12^. Planktonic dispersal patterns of paralarvae in an Iberian-Canary Upwelling system show larvae of *I. coindetii* within the group using «coastal strategy»^13^.

The Ommastrephidae are also the most important cephalopods for commercial fisheries^12^. During 2010–2014, the annual world catch of ommastrephids varied between 1.6 and over 4 million metric tonnes, representing 70% of the total world cephalopod catch (Jereb et al.^14^). Of these, the genus *Illex* contributes a significant portion, corresponding to 9.6% of the total annual cephalopod catches in 2015^15^. However, it is difficult to obtain specific statistics due to systematic uncertainties in the definition of each species and a common tendency of landing mixed squid species, and reporting even single species merely as “short-finned squid”.

Cephalopods have highly variable reproduction strategies^16^. Generally, cephalopods are short lived and semelparous^17^. Many observations indicate that *I. coindetii* females are “intermittent spawners” that spawn several times during a period of time ranging between a few days and a few weeks^12^, with a so-called intermittent terminal spawning pattern where oocyte maturation and egg-laying occur separately during the spawning period, which may last for several weeks^18^. The female does not grow in this period and dies shortly after. Statolith data indicate an average life span of one year or less^19–22^. Fertilization in ommastrephids takes place while spawning^23–25^. Knowledge on egg and juvenile development for *I. coindetii* is limited^11^, and egg masses have never been observed in situ except by Naef^2^ who reported *«floating devices»* from the Mediterranean Sea, together with his drawings of juvenile squid, later thought to be *I. coindetii*^26^. However, in vitro fertilization has been accomplished for *I. coindetii*, and hatchling morphologies have been described^27^.

The aim of this study is to determine which cephalopod species is responsible for the production of huge gelatinous spheres from NE Atlantic Ocean, using DNA analysis on sphere tissue samples. We also report on 90 huge spheres from the NE Atlantic extending ~ 35 years back in time (from ~ 1985 to 2019). Of these 90, 27 are reported in Ringvold and Taite^1^ and 63 are newly reported herein.

## Material and methods

### Collecting tissue samples

As a continuation of the study in Ringvold and Taite^1^, popular press articles and posters were published to obtain as many new sphere observations as possible, with associated ecological information, and to solicit collection of tissue samples for molecular identification. We focused these solicitations on Facebook Internet pages for diving clubs and diving centers in Norway, Sweden, England, Scotland, Ireland, France, Spain, Portugal, Italy, Malta, Cyprus and Croatia.

**Figure 1:**
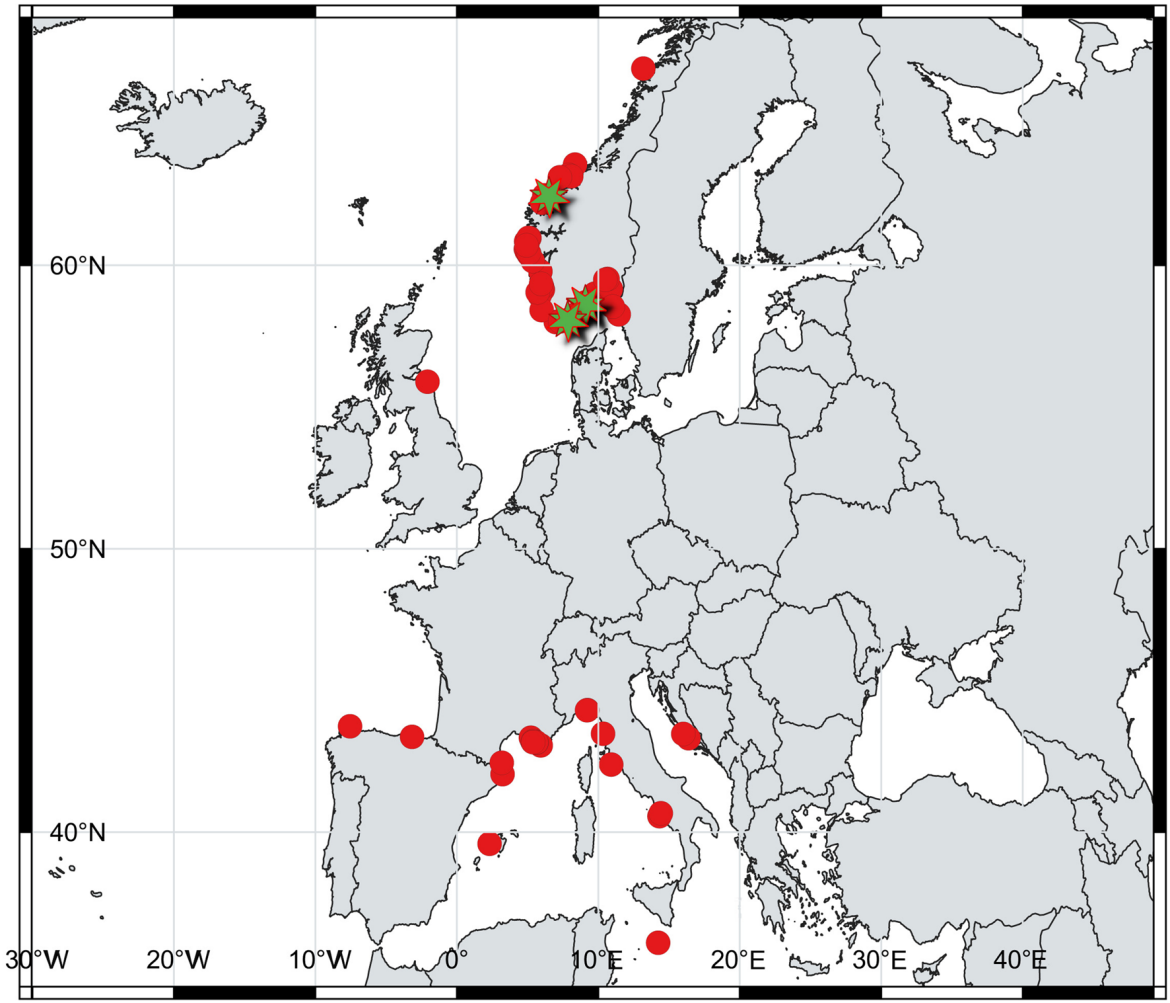
Locations where huge gelatinous spheres have been observed in the NE Atlantic Ocean, including the Mediterranean Sea (red dots), combining data from this study and Ringvold and Taite (^1^, Fig. 2). Locations of DNA tested spheres (green stars). Figure generated with Qgis 3.2. (www.qgis.org) (Credit: Halldis Ringvold/Sea Snack Norway).

On 28 July 2019 at noon two experienced technical Scuba divers at Magerholm in Storfjorden near Ålesund, NW Norway, encountered a large gelatinous sphere floating at 43 m depth, 2 m above the sediment (Table 1, Fig. 1). The sphere measured approximately 1 m in diameter, and had a narrow streak, or structure, through the center. One diver had a 0.5 l plastic bottle with screw cap. Because one of the divers had heard about our citizen science project through media, and our desire for a tissue sample, they agreed to take a sample using the plastic bottle. After squeezing out the freshwater, the bottle opening was placed underneath the sphere, sucking out some tissue. Directing a flashlight towards the bottle confirmed that eggs from the sphere was successfully collected. The sample was secured at 12:45, and in situ water temperature was 8 °C, measured with a Shearwater Petrel computer. The sphere did not seem to be damaged by the sampling but kept the same spherical shape as it drifted off. No picture of this sphere was taken.

**Table 1:** Synopsis of gelatinous sphere observation data from NE Atlantic Ocean, including the Mediterranean Sea, ~ 1985 to 2019.

Nr	Year	Date	Location	Latitude	Longitude	Time	Depth (m)	Temp (°C)	Size (m)	Streak through center	Comments from observers	Observed by	Diving club
**From Norway and Sweden**													
1	1995	spring/summer/fall	Storestongi, Outer Fensfjord, Hordaland County	60.836830	4.897126		15–20			?	Looked like a big balloon. A long time since the observation, so minimal details	Ørjan Solheim	
2	2001	27.05	Møvik strand near Kristiansand, Vest-Agder County	58.095844	8.000793	12:00	~ 10	15	1	Yes, black, rectangular structure inside	Observed on sand close to rock wall	Siri Kristoffersen	Private dive
3	2002	03.08	Fosnstraumen at Radøy, Hordaland County	60.729144	4.975777	12:00	~ 20	~ 11	1	Yes	Floating just above the sediment	Tom Christer Fløgstad, Geir Johannessen	Studentenes undervannsklubb Bergen
4	2004	14.07	West of Tyskerholmen at Askøy, Hordaland County	60.405503	5.114479	16:00	14		1.5–2	No	Floating. No visible structures inside	Arne Fredrik Steimler	
5	2004	17.08	Øygarden, between Straumsund and Osund, Hordaland County	60.57245	4.87587	10:48	28		1	Yes	Floating 1.5 m above sediment	Anette og Roy Ægir Jolma-Stensland	Stavanger dykkeklubb
6	2005	August	Inlet to Eidsfjorden, Sogn & Fjordane County	60.960569	5.125809	20:00	14	17	2	Yes	Floating in weak currents inwards the fjord. One diver, measuring 1.90, was 10 cm shorter than diameter of sphere	Three guests at Gulen Dive Resort	Gulen Dive Resort
7	2006	30.09	Utåker in Matrefjord, Hordaland County	59.777399	5.907211	12:00	15		1,.5	Yes	Sphere with a few lumps on the outside. Floating up towards to the surface after a while	Erling Svensen, Rudolf Svensen	
8	~ 2006		Korshamn near Lyngdal, Vest-Agder County	57.992459	6.990051						Information from Facebook-group	"krissk2"	
9	2006?	Summer/autumn	Langesund, Telemark County	58.995874	9.761656						Mentioned at www.nrk.no, 26.10.2006	Unknown	
10	2006?	Summer/autumn	Hitra, Sør-Trøndelag County	63.550485	8.352611						Mentioned in www.nettavisen.no in 2006 and at www.nrk.no, 26.10.2006	Unknown	
11	2008	09.08	South of Teistholmen, Stavanger, Rogaland County	58.967319	5.861549		~ 15			No	A possible sphere	Camilla Klippenberg, Håkon Sevheim	
12	Between 2008 and 2010	Unknown	Sørskår in Årdalsfjord, Rogaland County	59.135334	6.074503		22		~ 2	Yes	Sphere standing on sandy bottom in bay at Sørskår. Oval ball roughly 1.80 m high and 1 m wide	Rudolf Svensen, Leif Bruntveit	
13	2010	28.07	Hommersåk, Stavanger, Rogaland County	58.932906	5.847693		ca. 20		30 cm wide, 50 cm tall	No, but two spots	Resembles an "egg". Could be a possible sphere	Sine Trodal	
14	2010	11.08	Tustna, Kristiansund, Møre & Romsdal County	63.184414	7.893752	13:17	15	13–14	0.8	Yes	"Flexible sphere", observed while freediving. Air temperature 19 C	Tapio Salakari, Pyry Routakorpi	Archipelago Divers Association
15	2015	spring	Mæbøfjorden, Flekkerøy, Kristiansand, Vest-Agder County	58.059128	7.99736		3	10	0.3	No	A small sphere observed from pier, laying on bottom. Appearing soft and neutral in the water	Fridtjof Karlsen	
16	2016	~ 17.07	Tustna in Aura, Møre & Romsdal County	63.138416	8.084729	14:30	37	14	0.8–0.9	Yes	Floating sphere	Carl Ziegler, Kim Davidsson	Dive Tustna, Dykeriet
17	2016	17.08	Lille Torungen in Arendal, Aust-Agder County	58.412109	8.794769	18:30	37	14	~ 1	No	In week current	Geir Eliassen	Arendal undervannsklubb
18	2017	27.06	Tingelsete in Egersund, Rogaland County	58.413133	5.988922	16:00	10	10	1.5	Yes	Floating in area with dense kelp forest	Erling Svensen	
19	2017	06.07	West side of Foldnes at Fjell, Hordaland County	60.381526	5.082501	14:30	15–20	10	2	Yes	Floating southward. No other structures to be seen (but bad visibility)	Magnus Garberg	
20	2017	13.07	Kjeahålå farm at Ombo Island, Rogaland County	59.230634	6.024793	12:40	18	~ 15	2	Opaque ball, so did not see	Opaque sphere, like an egg. Three other spheres observed during ~ 13 years time, within 1 km. Some seemed empty. The spheres bump into the net on aquaculture farms. Neutral boyancy	Mantas Kaciulis, Stanley Moen	Fiskaa Undervannsservice as
21	2017	~ 17.07	Lysekil, Västra Götaland County, Sweden	58.261561	11.441217						Information from facebook-group	Rickard Larsson	
22	2017?	?	Lysekil, Västra Götaland County, Sweden	58.261561	11.441217						Information from facebook-group	Rickard Larsson	
23	2017	18.07	Sandnesfjorden/Lagfjorden near Risør, Aust-Agder County	58.68625	9.08672	16:19	0.5	18	2	Yes	Photographer saw it at surface from boat but ran into it with propellar before pictures were taken	Espen Danielsen, Felix Nordgaard Danielsen, Jan Petter Røseth	
24	2017	26.07	Rongsundet, Hordaland County	60.499904	4.929782	14:00	25	11	~ 1	Yes	In strong southern currents	Bjørnar Nygård	1Dykkeklubb
25	2017	28.07	Volsdalsberga in Ålesund, Møre & Romsdal County	62.466106	6.179501	15:30	17	10	> 1	No	The sphere seemed empty	Ronni Bless Bekkemellem, Torbjørn Inge Flor	Veterandykk Molde
26	2017	20.08	Ærøya at Arendal, Aust-Agder County	58.415197	8.768480	12:00	18	15.7	1	No	Currents 1 m/s. Lumps on the outside of sphere, and green algae hanging from it	Harald Pedersen	Arendal undervannsklubb
27	2017	30.08	Valøyene near Grimstad, Aust-Agder County	58.321526	8.616371	10:30	2	16–17	0.75	Possibly	Inside the sphere was one area slightly darker than the rest	Ola Brandt	
28	2017	02.09	Boat harbour at Åmøy, Rogaland County	59.042882	5.707644	13:00	surface	~ 15	Unknown	Probably	Possible sphere, but damaged, seen at the surface from boat. Surface currents 0.5–1 m/s inside sheltered harbour	John Johannessen	
29	2017	03.09	Jonsgrunnen in Meløy, Nordland County	66.940686	13.180847	09:00	2–3		0.75–1	No	Seen when fishing, in strong northwards currents	Synnøve Kåseth	
30	2017	~ 07.09	Kallsøyna, SW of Askøy, Hordaland County	60.459328	5.028305		Fishing net at max. 70 m	14	0.7	No	Caught in fishing net	Terje Vindenes	
31	2018	June	Lillesand area (?), Aust-Agder County	58.225407	8.401089						Several spheres have been spotted, but sizes and dates unknown	Lise and Peter Walker	
32	2018	10.07	Kråkerøy, Enhuskilen, Østfold County	59.154748	10.882782	12:00	surface	17.5	0.4	Yes		Trine Elisabeth Hobøl	
33	2018	11.07	Trolleskär, Sweden	58.536941	11.019656		8	14	0.5	Yes		Birgitta Lantto, Zoltan Mate	
34	2018	21.07	Os, Strøneosen, Hordaland County	60.145565	5.364418		3		0.6–0.7	?	Spotted while freediving	Arne Indrearne	
35	2019	10.07	Between Barstadvika and Festøy, Ålesund, Møre & Romsdal County	62.363402	6.280919	~ 21:45	13	~ 12	1	No	Transparent, seemed empty and entangled into kelp	Harald Woldsund, Pauline Neergård, Margunn Alice Nordli	OSI dykking
36	2019	17.07	Dalsholmen, Arendal, Aust-Agder County	58.516770	8.925106		12	12	0.7	Yes	Tried to catch the sphere using liftbag, but the sphere dissolved. It looked like an old sphere	Geir Eliassen	Arendal undervannsklubb
37	2019	18.07	Bufjord camping, Homborsund, Aust-Agder County	58.282375	8.523008		1.5		0.4	No	Spotted from shore	Evelyn Aagestad, Gro Magnhild Fosteråsen, Siri Karlstorp Rustad	
38	2019	19.07	Nordnes, Foldøy, Rogaland County	59.33988	5.95540		5	~ 12	~ 0.4	No	Sphere had a "tale" and two "ears". Observed close to a wall	Tor Erik Meland	
39	2019	19.07	Lillesand, Aust-Agder County	58.225407	8.401089							Peter Walker	
40	2019	21.07	Buholmen, Kristiansund, Møre & Romsdal County	63.102972	7.27572	11:39	~ 7	10	> 1	Yes	Transparent sphere was laying on sandy bottom	Bjørn Sem	
41	2019	21.07	West of Mandal, Vest-Agder County	58.018268	7.304581	21:00	10	16	1	No	A probable sphere (collapsed shape), was observed	Pim Midling, Erik Wang	
42	2019	23.07	Gulafjorden, Sogn & Fjordane County	60.963970	5.066112	15.45	20	12	0.8	Yes	Observed 2 nautic miles from Gulen Dive Resort	Guests	Gulen Dive Resort
43	2019	28.07	Magerholm, Storfjorden, Ålesund, Møre & Romsdal County	62.429352	6.510029	12:45	43	8	1	Yes	A narrow streak through center of sphere. **TISSUE SAMPLE PROVIDED.** Technical divers	Peter Gundelfingen Olejar, Martin Hansen	Oceanfjord as, Ålesund sportsdykkerklubb
44	2019	28.07	Between Småværa and Hauglandsøy, Askøy, Hordaland County	60.432810	5.084742		15–20		0.5	Yes	Free floating	Morten Gjellestad, Grzegorz Marsicki	Askøy sportsdykkerklubb
45	2019	01.08	Volsdalsberga in Ålesund, Møre & Romsdal County	62.466106	6.179501	16:40	19	13	0.8	A darker part	A larger dark area on one side of sphere, narrowing towards the center	Andreas Olsson, Leif Haagensen	Ålesund sportsdykkerklubb
46	2019	07.08	Kilsund, Arendal, Aust-Agder County	58.580278	9.082500	14:54	17	14	0.5	Possibly a vague streak seen on video	Deformed, dissolving sphere. **TISSUE SAMPLE PROVIDED**	Geir Eliassen, Anita Eliassen	Arendal undervannsklubb
47	2019	08.08	Kilsund, Arendal, Aust-Agder County	58.611667	9.084722	10:15	15	15	0.8	A short streak	Small eggs inside sphere. Several lumps on the outside. **TISSUE SAMPLE PROVIDED**	Geir Eliassen, Anita Eliassen	Arendal undervannsklubb
48	2019	11.08	Våtmyrholmen, Sykkylven, Møre & Romsdal County	62.400777	6.481842	14	11	12	1	Possibly a darker, short area	Not quite spherical. "Fluffy" consistency when waving hand above sphere	Robert Fiksdal, Thomas Jenssen	Ikornnes dykkerklubb
49	2019	13.08	Bøvågen, Radøy, Hordaland County	60.701945	4.920791	11:30	16	10	04–0.45	No	Observed during wreck diving. Lump on the outside	Grzegorz Marsicki	Askøy sportsdykkerklubb
50	2019	16.08	Ny Hellesund, Søgne, Vest-Agder County	58.052542	7.832505		10	15	0.5	No	Decaying sphere. On bottom, trapped underneath kelp. **TISSUE SAMPLE PROVIDED**	Karl Klungland	Søgne dykkerklubb
51	2019	24.08	Kalvøysund, Kristiansand, Vest-Agder County	58.247500	8.340833		20		0.7–0.8		Sphere oval/sphere shaped, floating free in water mass. Thousands of embryos in sphere	Geir Eliassen, Anita Eliassen	Arendal undervannsklubb
52	2019	25.08	Bevøya, Moss, Østfold County	59.514036	10.647280	12:30	20	14–16	0.4	2 red/dark dots inside	Whole sphere floating about 0.3 m above sandy, muddy sediment	Tommy Pedersen	
53	2019	24.08	Homborsund lighthouse, Aust Agder County	58.255147	8.513636	18:00	0.5	~ 18	0.3–0.4	One dark dot	A floating sphere was spotted at 0.5 m depth, when freediving. Total depth at site was 3 m	Roald Andreassen	
54	2019	12.09	Storfjorden, Ålesund, Møre & Romsdal County	62.433544	6.522615		23	13			Possible remains of a dissolving sphere: a gelatinous flake (25–30 cm in length) entangled in kelp, with 2–3 possible eggs/embryos, about 4 mm in length. Diver has previously seen a whole sphere	Martin Hansen	Oceanfjord as
55	2019	05.10	Ørstadfjorden, Møre & Romsdal County	62.202394	6.026276	10:30	17	10	~ 0.9	Yes	No currents, so sphere was not mooving. Neutral in water. Some freshwater down to approx. 25 m depth	Ronald Raasch, Nils Baadnes, Brynjar Aarnseth	Frosta Froskemannsklubb. Levanger Undervannsklubb
56	2019	20.10	Mølen, Hurum, Oslofjord, Buskerud County	59.486899	10.49111		30	9	0.6–0.7	No	Free floating, transparent sphere	Bjørn Sem	Moss undervannsklubb
**England/Scotland border**													
57	2016	August	East of Eyemouth, 30 miles off shore, NE England	55.884847	-2.080327		18	7–10	1–1.2	Dark mass in the middle	Sphere drifted past divers whilst on deco stop. Roughly spherical but not round. One diver poked it and it was firm. Three technical divers with 25–30 years of experience each sees sphere for the first time	Steve Burke, Steve Saunders, Lorne Thomson	Diving with Marine Quest/Iain Easingwood at wreck of NJ Fjord
**From Spain, Atlantic coast**													
58	1985/1986		Ontón, Cantabria, Spain	43.35881	-3.170757		6–8	16–18	~ 2		Floating sphere	Patxi Berastegui	
59	2017	24.03	Ensenada "Fontans", near Viveiro, Galicia, Spain	43.731616	-7.553307	~ 17–19	10	15	1	yes	Sphere observed while floating over rocky bottom, and after a while it seemed wedged into a small ditch	Eduardo Losada Lage	
**From Mediterranean Sea**													
	1900	September	Naples, Italy				surface				Adolf Naef observed ommastrephid larvae "on floating spawn" in Naples, in 1900. This "spawn" was not illustrated	Adolf Naef (Ref.^2^)	
60	1999?	October	Island of Brac, Milna, Croatia	43.309411	16.380066		53		0.8	Yes		Miro Andric (Ref.^28^)	
61	2005	18.09	Farillons, off Marseille, France	43.207798	5.338961		30		1	Yes	An illustration of a sphere with a streak through center has been drawn. Coral-colored tube, expanding at each end, is crossing the center of the sphere	Christine Baudin	
62	2006	September	Muljica, near Otok Arkandel, Croatia, Adriatic Sea	43.463396	16.00296		50	17	1.5	Yes	Transparent and free floating sphere	Borko Pusic, Roy Soage, Marko Prasek	Diving Center Pongo, Croatia
63	2009	22.08	Castel viel, Cassis, France	43.197437	5.503670	09:00	45	20	1	Yes	In suspension 50 cm above a sandy bottom	Alain Beauté	Pertuis France
64	2010	22.08	Portofino, National park, close to Secca dell'Isuela, Italy	44.303321	9.225220	~ 10:00	33	~ 24	0.8–1	Yes	Bad visibility in the water	Simone Ulzega, Francesco Litrico	
65	2011	23.04	Punta Campanella, Gulf of Naples, Italy	40.558684	14.319745	15:22	50	14	1	Yes	Resting on the bottom. Spotted on a plateau, close to a wall	Edoardo Ruspantini, Daniel Castrucci	Punta Campanella Diving
66	2011	31.07	Ile de Riou, Calanques, Marseille, France	43.171411	5.394122	10:00	25		1	Yes	Sphere was floating a few meters above the seafloor	Thomas Brelet, Carole Baffert	L'enfant et la Mer
67	2013	22.06	Les Deux Fréres, Saint Mandrier, France	43.056757	5.925712	15:47	6	19	0.80	An oval, dark spot	Floating	Renauld Helstroffer	Thalassa
68	2013	08.08	Cap Caveau, Marseille, France	43.260047	5.288527		4		0.6–0.7	?		Francois Savineau	
69	2013	26.08	Ile verte la Ciotat, France	43.158489	5.620880	morning	24	14	0.7–0.8	No?		Perrine Moreau	
70	2013	14.09	Ile de Riou, Parc des Calanques, Marseille, France	43.178432	5.389326		6	14	1.5	Yes	Top of the sphere full of white dots	Franc Jourdan, Alexandre Sassatelli	FCSMP
71	2013	17.09	Pointe Cacau, Cassis, France	43.197212	5.510244		21	14	0.75	Yes	Strong winds	Fabienne Henry	Narval Plongée
72	2014	15.08	Le Mejean, Frapao, France	43.328998	5.230146		~ 10			Yes		Sabine Boulad, Audrey Joulia	
73	2014	15.08	Le Mejean, Frapao, France	43.328998	5.230146	11:00	~ 5	> 22	1	Yes	The sphere was partly opened at one end	Edouard Bard	Plongée Passion Carry
74	2014	21.08	Archipel de Riou, Pointe de Caramaseigne, France	43.175653	5.398853	09:50	45	17	1.2	Yes	Observed for five minutes. When approaching the sphere with the hand, the sphere shape changed form due to water pressure. When removing the hand, the shape went back to original shape	Philippe Le Roy	Atoll de Marseille
75	2014	12.09	Reqqa point, Malta	36.090098	14.227787	13:40	52	21	1	YES	Free floating sphere moving with slow current	Sonia Silvio, Jeffery Falzon	Atlantis Diving Centre
76	2015	02.05	Pointe Cacau, Cassis, France	43.197421	5.509678	morning	5		1	Yes		Frédéric Di Meglio	
77	2015	31.07	La Ciotat, France	43.163760	5.610688		3		1.5	Yes	Two black "spots" inside	Jean-Pierre Croce	
78	2015	02.09	Ile verte La Ciotat, France	43.158489	5.620880		25		1–1.5	Yes		Claude Chanteux	
79	2015	05.09	Castel Viel, Cassis, France	43.197437	5.503670	morning	30 or ~ 40?	19	1–1.5 (?) [sic]	Yes	Several divers saw the same sphere	Sandrine Clerc, Jackie Dozin, Denis Baranger via Christine Lacouture and Alain-Pierre Sittler	Narval Plongée
80	2015	06.09	Sormiou, near Marseille, France	43.209435	5.402801	15:00	3	15	1.8	Yes	The sphere had a black string in the center. A hole was present on the surface of the sphere (like a torn tissue). The sphere was not moving	Rémy Guillo du Bodan	Private snorkeling
81	2016	08.05	National Park, Portofino, Italy	44.298210	9.200500	11:00	~ 12	16	1.0–1.2	No		Pietro Crovetto	
82	2016	23.06	Medes Islands, Tascons Petits, Estartit, Spain	42.041456	3.226798	11:30	18–20	20–21	1	No	Transparent with lots of "lobes"—no visible streak inside. Seemed empty. Recorded 10 m from a wall	Elisabeth de Longeville, Christine Chesnay	Sensation Paris Plongée
83	2016	02.08	La Ciotat, Le Mugel, France	43.163760	5.610688	10:11	17		0.6–0.7	Yes		Emmanuel Roguet	
84	2016	06.08	Les Moyades, Marseille, France	43.176495	5.370707	15:55	20	14	1.2	Yes	Spotted day after strong Mistral (wind from the south of France), in calm sea	Cyril Feuillet, Jean Marie Perrin, Patrick Carreno	Private dive
85	2017	25.04	Calafuria, Italy	43.469198	10.331749		30		1	No?	Possibly egg mass of *Ommastrephes bartramii*	Fabio Benvenuti, Luigi Macchi	
86	2017	27.05	Cerbére, France	42.445413	3.173058		11		0.3	Blackish dot		Natalia Vazart	
87	2018	13.08	Isla Dragonera, Balearic Island, Mallorca, Spain	39.583333	2.309444	11:15	48	16.5	1.4	Yes	Spotted 1 m from the bottom	José Coronel	ZOEA Mallorca Diving Center
88	2018	06.09	Isola del Giglio, Secca della Croce, Toscana, Italy	42.380842	10.910591	afternoon	~ 60	~ 15	1.2–1.5	Yes	Spotted 40 cm above sediment, in weak currents. Technical divers	Carlo Lorenzetti	
89	2019	24.03	Seiano, Gulf of Naples, Italy	40.663052	14.418457		5–6		0.3–0.4	?	Spotted while freediving	Rosella Brivio	Narval Plongée
90	2019	30.09	Impériaux (Riou island), Marseille, National Park of Calanques, France	43.171719	5.39383	morning	45	20	0.45	Yes	Twisted, whitish streak	Charly Roba, Jonathan Mouton	Narval Plongée

The data from this study are combined with data from Ringvold and Taite^1^. Estimated size in diameter, and latitude and longitude in decimal degrees, DD (approximate locations). (Records by Adolf Naef^2^ is not included in count of spheres.) * Observations 73 and 74 might be the same sphere.

When ashore the bottle was transported to the home of one of the divers, in an air conditioned car, and placed in the freezer. The next day transport was arranged for the sample to the nearest research station (Møreforsking in Ålesund) 10 min away, and the bottle contents were thawed after arrival at the station.

At the laboratory the bottle contents were poured into a 1 l sterile beaker. The first attempt to separate eggs from their mucous matrix led to the pipette being clogged with slimy mucus. The bottle contents were therefore poured into several petri dishes, and the eggs were successfully pipetted into seven 15 ml tubes. A ratio of about 2 ml of eggs and some gelatinous matrix to 13 ml 96% ethanol was used, and eggs were visible in the ethanol solution. Two eggs were preserved in a 4% formalin solution. Photos of a few thawed elipsoid eggs were taken with a handheld mobile camera through a microscope. Thawed egg length was estimated by eye. After making sure that all eggs had been extracted from the sample, the excess seawater and gelatinous matrix was transferred to five 50 ml tubes with 96% ethanol, which were stored cool (4 °C). All samples were shipped immediately to Sea Snack Norway (< 24 h transport).

On 7 and 8 August 2019 two additional sphere tissue samples were obtained near Kilsund in Arendal, SE Norway, at 17 and 15 m depth, respectively. The first sphere, collected 7 August, was disintegrating with the sphere wall rupturing. Tissue was secured in a small plastic jar with screw cap. After returning to the surface, the divers transported the jar to a home freezer (approx. − 20 °C). A few days later, it was delivered to Flødevigen. When thawed, only a small part of the sample was left in the jar. The sample seemed to be a part of the outermost section of the sphere, containing a mix of gelatinous matrix and algae. The sample was investigated in a petri dish under the microscope and a single developed embryo was found and measured.

A sample of the second sphere, collected 8 August, was secured in a 0.5 l plastic bottle with screw cap, using the same method as for the Ålesund sample, and delivered to the nearest research station, Institute of Marine Research at Flødevigen the same day. The eggs could be seen floating in the mass of clear gelatinous matrix mixed with seawater. The volume of gelatinous matrix was estimated as 2–3 deciliters. The sample was poured into a big petri dish, put under a dissecting microscope and live eggs were isolated from the sticky egg mass using a dissection needle and a disposable pipette cut to a suitable size. The eggs, about 65 in total, were transferred to a small staining jar and investigated further under the microscope. Pictures of live embryos were taken through the eyepiece of the microscope. The size of the embryos was also measured and checked with a calibrated metal sphere of 1.00 mm.

Tissue from both spheres was preserved directly in 96% ethanol. A few eggs collected on 8 August were preserved in 4% formalin, and a few were conserved in separate tubes with 96% ethanol, for possible additional analysis (not herein).

On 16 August 2019, tissue of a fourth sphere was collected at Søgne, at 10 m depth. The diver secured a sample of a dissolving sphere using a 0.5 l plastic bottle with screw cap, and when at home the sample was placed in the freezer. A few days later sample transport to University of Agder in Kristiansand was arranged. After defrosting, the content of the bottle was placed in petri dishes and analysed through a Zeiss Stemi DRC stereo microscope with ocular micrometer scaling slide using 16 * ocular and 1.6 * object zoom, where 10 sublines corresponded to 0.55 mm. Pictures were taken using an iphone 7 mobile camera through the eyepiece of the stereo microscope. Whole embryos and most pieces of embryo in the sample were pipetted out and conserved in 96% ethanol for DNA analysis. A few embryos were also preserved in 4% formalin. All samples were shipped to Sea Snack Norway.

Ethanol-preserved tissue samples of adult *Todarodes sagittatus* (Lamarck, 1798) were provided from cruises by Faroe Marine Research Institute (Faroe Islands) and Marine & Freshwater Research Institute (Iceland). Ethanol-preserved tissue samples of adult *Illex coindetii* were provided by Stavanger Museum.

All ethanol samples were shipped from Sea Snack Norway to University of Ireland, Galway, for DNA analysis. Voucher samples are deposited in the collections of Stavanger Museum, Norway.

### Other observations

DORIS (Données d'Observations pour la Reconnaissance et l'Identification de la faune et la flore Subaquatiques) is a citizen science project initiated in 2006 by the “environment and biology commission” of the French Underwater Federation (FFESSM). The aim was to construct a web site presenting French aquatic species (including French overseas territories). More than 2.600 species are described and illustrated by about 26.000 pictures. The DORIS network was contacted by Professor Helmut Zibrowius (Marseille Oceanology Centre) in 2007, who asked about observations, photos, and samples of an unknown huge sphere observed in Croatia in 2006. The huge sphere was named GST (“Grande Sphère Transparente” i.e. huge transparent sphere). The DORIS network enabled recording of sphere observations from France more than 10 years back in time—to the present; these observations are included in this study. Unfortunately, no sphere tissue samples have yet been taken from French, or Mediterranean, waters. One observation was also mentioned from Croatia, by Miro Andric^28^.

### Molecular analysis

The four gelatinous sphere tissue samples collected from Norwegian waters were well preserved in ethanol and eggs were clearly present, some with visible embryos. DNA was extracted from the eggs from the spheres, as well as from separate tissue samples of adult *T. sagittatus* and *I. coindetii,* using a Purelink genomic DNA mini kit following the manufacturer’s instructions. Universal Folmer primers LCO1490 (5′-GGT CAA CAA ATC ATA AAG ATA TTG G-3′) and HCO2198 (5′-TAA ACT TCA GGG TGA CCA AAA AAT CA-3′)^29^ were used to amplify the Folmer region of the cytochrome oxidase subunit I (*COI*) gene. Each PCR contained 12.5 μl DreamTaq Green PCR Master Mix (Thermo Scientific), 0.5 μl of each primer (10 μM), 9 μl nuclease-free water (Thermo Scientific) and 2.5 μl DNA template (20 ng). The PCR thermocycling program included an initial step at 94° C for 2 min, followed by 35 cycles at 94 °C for 40 s, 50 °C for 40 s and 72 °C for 90 s which was followed by a final step at 72 °C for 10 min. The sample was assessed by electrophoresis on a 1% agarose gel stained with SYBR Safe DNA Gel Stain (Invitrogen, LifeTech). PCR products were purified using the Purelink PCR purification kit following the manufacturer’s instructions and sequenced by GATC Biotech (Constance, Germany) on a Sanger ABI 3730xl.

BLAST^30^ indicated that the DNA extracted from the spheres probably originated from *Illex* sp.. Thus all available *Illex COI* sequences were downloaded from GenBank^31^, excepting those where the squid had not been identified to species using morphological methods (i.e., excluding studies which were themselves using DNA to identify species), those that were unusually short, and known contaminants (e.g., «*Illex argentinus*» from^32^ from a Korean fishmarket which are now known to have originated from *Nototodarus sloanii*). Downloaded sequences were combined in a fasta file with those sequences generated herein, and four additional sequences of *T. sagittatus* (also from Genbank). Sequences were imported to Unipro UGENE^33^, aligned with Muscle. The resulting alignment contained 73 sequences (13 new) and was 624 base pairs long. A maximum likelihood tree, rooted on the *Todarodes* sequences, was built in RAxML version 8^34^ using raxmlGUI 2.0 beta^35^. 100 fast boostraps (BS) were generated using the GTRGAMMA model and default settings.

### Ethical approval

In accordance to Norwegian and European legislation related to animal research, formal approval of the experimental protocol by the Norwegian Animal Research Authority (NARA) is not required because the experimental conditions are practices undertaken for the purpose of recognized animal husbandry. Such practices are excepted from the European convention on the protection of animals used for scientific purposes (2010/63/EU), cf. article 5d. Also, these practices do not require approval by the Norwegian ethics board according to the Norwegian regulation on animal experimentation, § 2, 5a, d “non-experimental husbandry (agriculture or aquaculture)” and “procedures in normal/common breeding and husbandry”. Norway has implemented the European Directive according to the EEA agreement. This explanation may be viewed as a waiver. The Norwegian Animal Research Authority does normally not give formal waivers. I.e. in clear cases, such as this one. Experiments involving in situ captured, fertilized squid eggs prior to exogenous feeding are exempted from the Norwegian Regulation on Animal Experimentation.

## Results

### Molecular analysis

**Figure 2:**
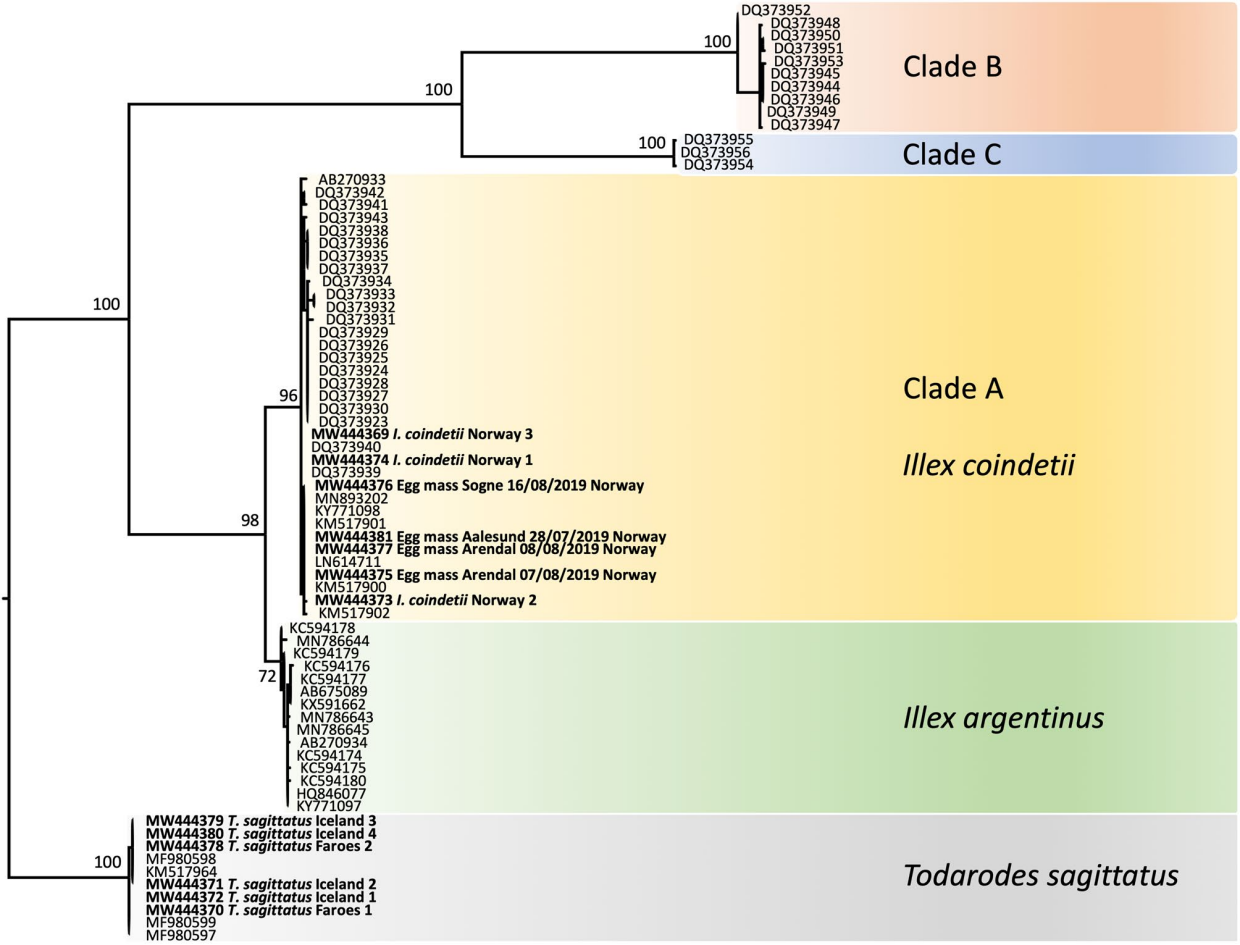
Maximum likelihood tree based on 624 base pairs of *COI*. Newly generated sequences shown in bold text.

The newly generated sequences are available through Genbank with Accession Numbers MW444369-MW444381. They include four *COI* sequences of egg masses, and additional *COI* sequences from adult squids as indicated in methods. The corresponding specimens are deposited in Stavanger Museum, Norway. All sequences from egg masses resolved in a highly supported clade (BS) together with new sequences obtained from specimens identified as *I. coindetii* from Norwegian waters (Fig. 2).

### Morphology

Embryos from four spheres where recorded, at four different embryonic stages. When describing stages, we follow Sakai et al.^36^.

### Ålesund sample

**Figure 3:**
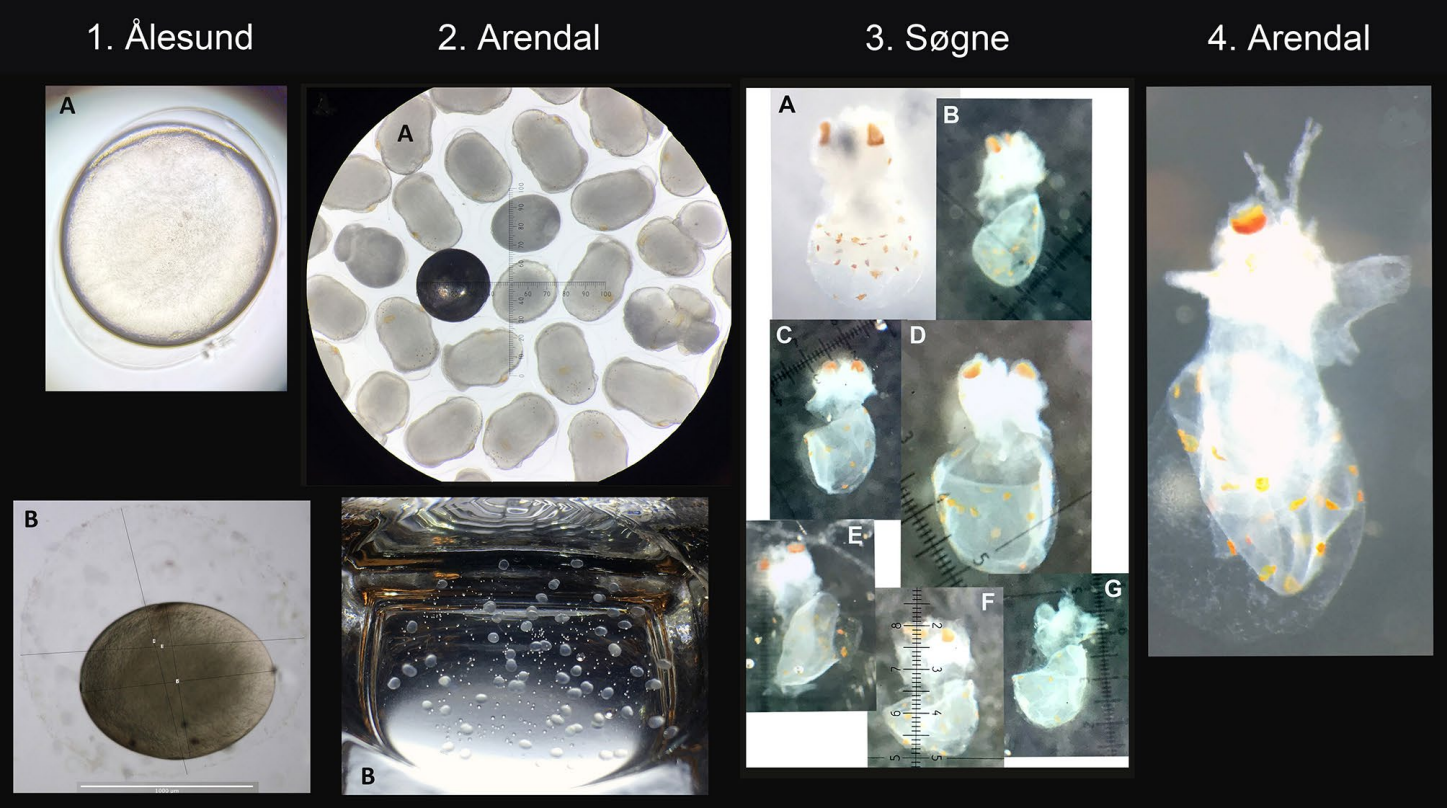
(1) Ålesund sphere sample (thawed): (**A**) Recently fertilized, elipsoid egg of *Illex coindetii* measuring between 1–2 mm in length (by eye). (**B**) Formalin fixated egg. Chorion length and width for one egg measured 1.13 and 0.91 mm, respectively, and the jelly envelope length and width were measured to 1.65 and 1.59 mm, respectively. (2) Arendal sphere sample: (**A**) Live embryos of *Illex coindetii*; 16 embryos measured width of 0.77 ± 0.047 mm and length of 1.15 ± 0.119 mm (from microscope pictures (collected 8 August). Black measurement sphere is 1 mm in diameter. (**B**) Arrangement of live embryos within the egg mass in the internal mucous matrix. (3) Søgne sphere sample (thawed): Seven developed embryos of *Illex coindetii*, of which five were measured, showing total length of 1.83 ± 0.139 mm (from microscope pictures). (4) Arendal sphere sample (thawed): Developed embryo measuring approximately 2 mm in length (by microscope) (collected 7 August). (Photo credits: Martin Hansen/Oceanfjord as, Snorre Bakke/Møreforsking, Arne Hassel and Hanne Sannæs/IMR, Ann-Elin Wårøy Synnes/University of Agder. Collage Halldis Ringvold/Sea Snack Norway.).

The sphere was whole, and when the diver poked it with his hand the consistency seemed firm. The gelatinous matrix and eggs from the Ålesund sample were all transparent and sticky. About 15–20 eggs were isolated, all approximately developmental stage ~ 3. Estimated egg length was between 1–2 mm (estimation by eye). All eggs were ellipsoid, and recently fertilized, with micropyle visible. The yolk sac, the embryo, and the chorion are visible at this stage (Fig. 3). The embryo has also covered the animal pole of the yolk with a layer a few cells thick and is spreading to cover the entire yolk. On the formalin-fixed egg (Fig. 3), the darkened chorion is visible in the jelly envelope. Chorion length and width for one egg were measured with a Leica stereomicroscope to 1.13 and 0.91 mm, respectively, and the jelly envelope length and width were measured to 1.65 and 1.59 mm, respectively.

### Two Arendal samples

The sphere sampled 7 August was disintegrating, and of loose consistency. The internal mucous matrix was sticky. A single developed embryo (around stage 30) was found, not in the matrix but in sea water associated with the sample. It measured about 2 mm in total length (Fig. 3, video 1).

The sphere sampled 8 August was whole and of firm consistency (video 2). Eggs were elipsoid, and both eggs and the internal mucous matrix were sticky. Embryos had reached organogenesis stages 17–19, and 16 of the embryos measured width of 0.77 ± 0.047 and length of 1.15 ± 0.119 (Fig. 3).

### Søgne sample

The sphere was dissolving (video 3) so much that when a hand was waved close to the sphere wall, it ruptured. The internal mucous matrix was sticky. Seven whole embryos were found, as well as a few more head fragments, from developmental stage ~ 23, and total length measured 1.83 ± 0.139 mm (Fig. 3).

In summary, the consistency of the four spheres varied, and changed from firm to disintegrating stage—as the embryos became more developed (Table 2).

**Table 2:** Similarities on spheres made by *Illex coindetii*: shape-, consistency- and diameter of sphere, embryo developmental stage, transparency level of sphere and sphere «health», based on tissue samples, pictures and videos.

	Ålesund	Arendal (8 August)	Søgne	Arendal (7 August)
Parameters				
Shape	Spherical	Oval/spherical	Ovoid	Long "scarf"
Consistency	Firm	Firm	Floppy, Decaying	Decaying
Diameter (m)	1	0.8	0.5	0.5
Embryo developmental stage	3	17–19	23	30
Transparency level	Transparent	Transparent	Opaque	Opaque
Sphere "health"	Good	Good	Bad, rupturing	Bad

### Observations of gelatinous spheres

**Figure 4:**
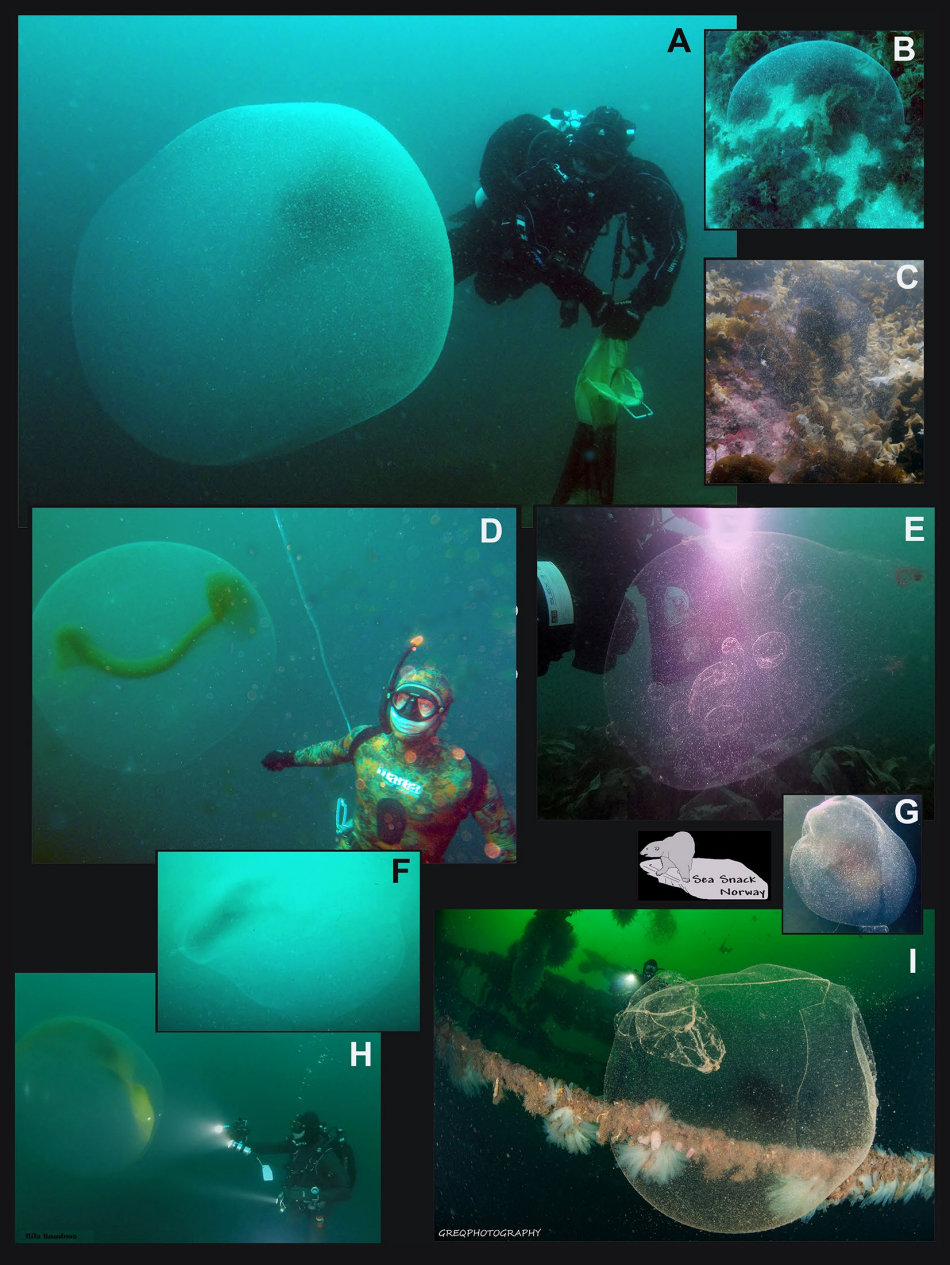
Huge gelatinous spheres from the NE Atlantic Ocean (Norway, Sweden and England) attributed to squid egg mass. (Photo credits: (**A**) Robert Fiksdal, (**B**) Harald Woldsund, (**C**) Birgitta Lantto, (**D**) Tapio Salakari, (**E**) Geir Eliassen, (**F**) Steve Saunders, (**G**) Tor Erik Meland, (**H**) Nils Baadnes, (**I**) Grzegorz Marsicki. Collage by Halldis Ringvold/Sea Snack Norway).

**Figure 5:**
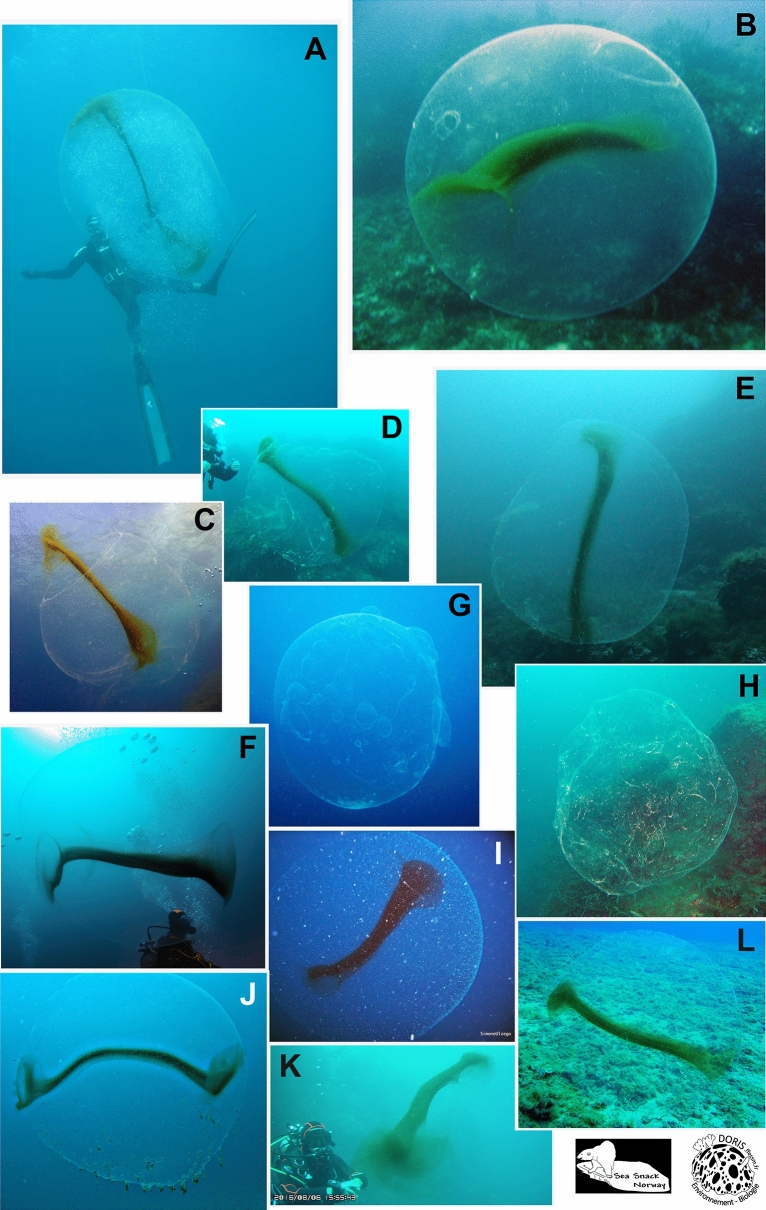
Huge gelatinous spheres from the Mediterranean Sea and the Spanish Atlantic coast, attributed to squid egg mass. (Photo credits: (**A**) Franc Jourdan, (**B**) Thomas Brelet, (**C**) Edouard Bard, (**D**) Eduardo Losada Lage, (**E**) José Coronel, (**F**), Philippe Le Roy, (**G**) Christine Chesnay, (**H**) Pietro Crovetto, (**I**) Simone Ulzega, (**J**) Alain Beauté, (**K**) Patrick Carreno, (**L**) Emmanuel Roguet. Collage by Halldis Ringvold/Sea Snack Norway).

Spheres have mostly been recorded from Norway and the Mediterranean Sea. In Norway, they have been recorded from Nordland County in the north to Østfold County in the south. Also a few spheres have been recorded from the Swedish westcoast (outlet of Gulmarsfjorden) (Fig. 4). In the Mediterranean Sea, they have mainly been recorded from the western basin (Spain, including Mallorca, France, Italy and Malta), but two observations are from Croatia (Fig. 5, video 4.). Since the study published by Ringvold and Taite^1^, observations have also been made in two new areas—NW coast of Spain (Bay of Biscay) (video 5) and NE of England, close to the Scottish border (Fig. 4).

**Figure 6:**
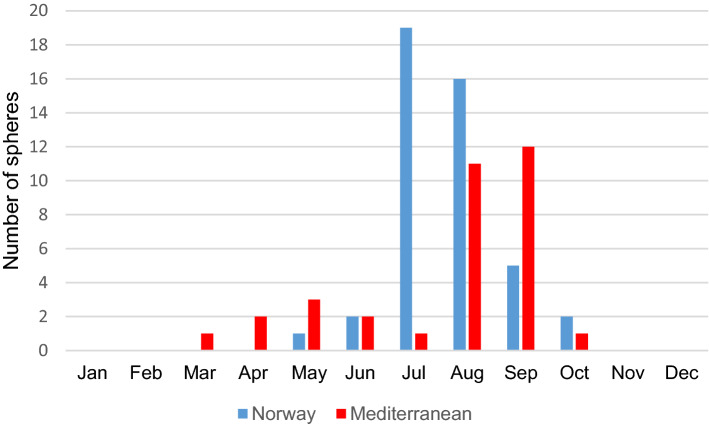
Sphere observations throughout the year, during ~ 1985 to 2019, from NE Atlantic Ocean, including the Mediterranean Sea.

**Figure 7:**
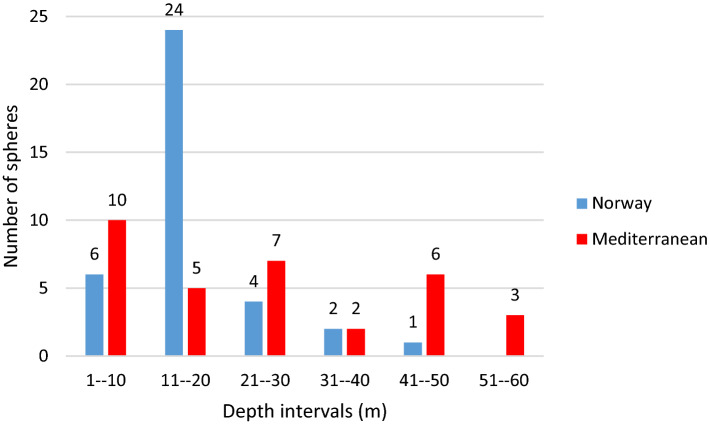
Sphere observations at different depth intervals, from NE Atlantic Ocean, including the Mediterranean Sea.

Spheres were observed from March to October (all areas combined) with a peak in July and August in Norwegian waters, and August and September in the Mediterranean Sea (Fig. 6). Based on all data we have compiled (Table 1), the egg spheres are divided into three sphere size ranges, < 0.5 m, 0.5–1 m and > 1 m in diameter. More than half (58%) of the spheres measured between 0.5 and 1 m in diameter. The depths of these spheres were from the surface to ~ 60 m (Fig. 7), but most spheres near Norway were observed from surface to 20 m depth whereas from the Mediterranean region most were from the surface to 30–50 m depth. Depths down to approximately 30 m are those visited by recreational sports divers.

**Figure 8:**
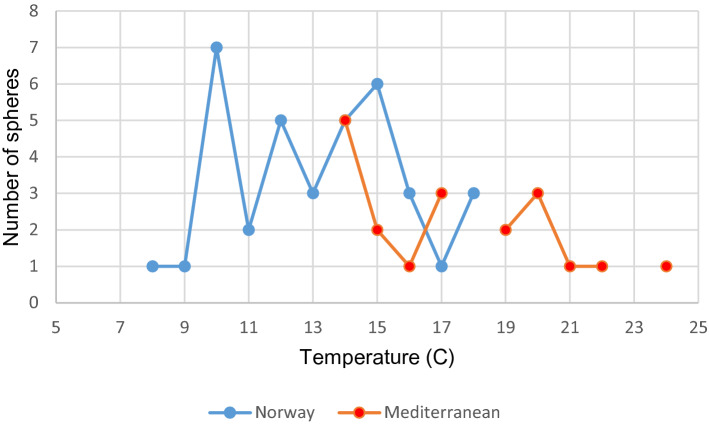
Sphere observations in different water mass temperatures, from NE Atlantic Ocean, including the Mediterranean Sea.

Most spheres were observed during daytime (all areas combined), but one is also observed during night diving. Night diving is a more rare activity. Temperatures ranged (for all areas combined) 8–24 °C. For Norway the temperature range was 8–18 °C, and for the Mediterranean region 14–24 °C (Fig. 8). Around 55% of spheres from all areas combined had a dark streak, or structure, through the center.

**Figure 9:**
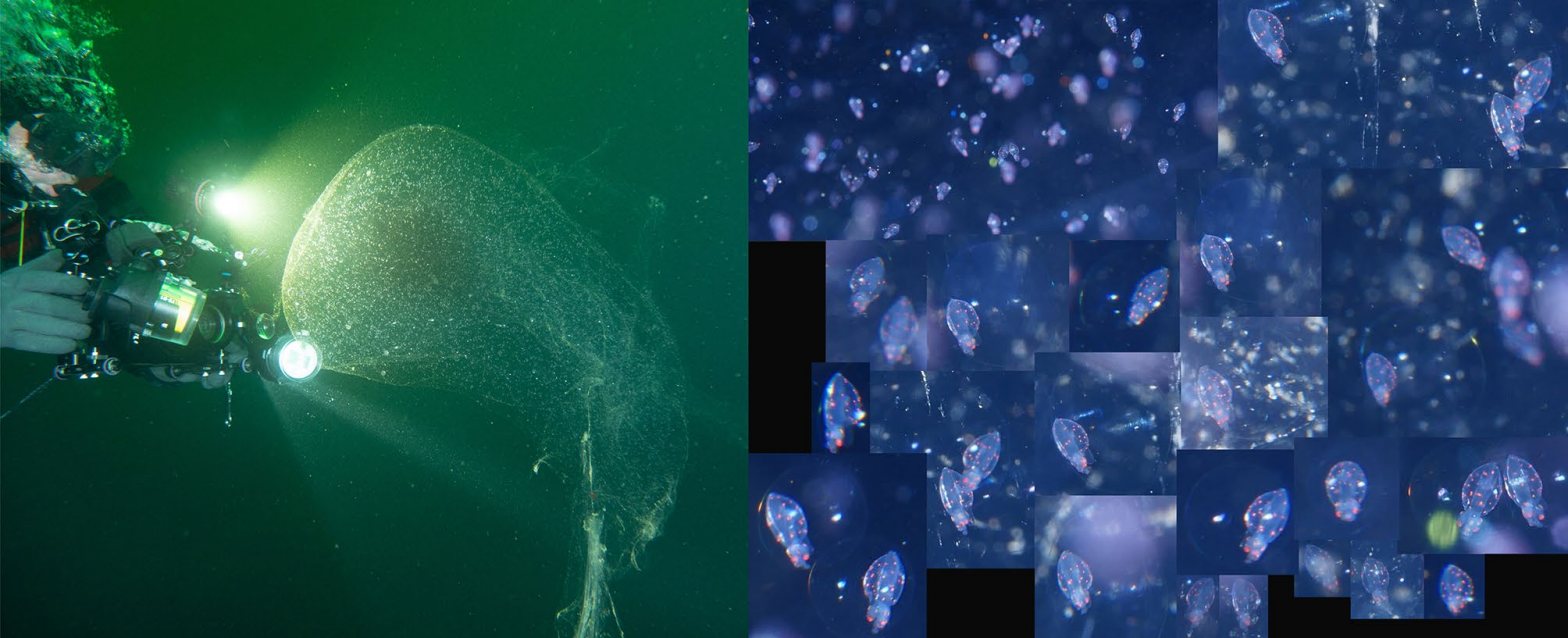
Whole sphere (deformed) with live embryos, photographed in situ from Kristiansand area (sphere observation 24.08.2019). Note the different orientation patterns of the embryos within the jelly envelope, with more or less all heads facing downwards. This sphere has not been genetically tested, but is high likely made by *Illex coindetii*. (Photo credits: Anita Eliassen (sphere) and Geir Eliassen (embryos)/Arendal undervannsklubb. Collage Halldis Ringvold/Sea Snack Norway).

In 2019 the same divers observed four spheres in the Arendal-Kristiansand area (S/SE Norway) from 28 July to 24 August (Fig. 9). There were also several reports from Lillesand to Mandal area the same year, as well as from Møre & Romsdal county (video 6). At an aquaculture farm in Stavanger area, Ombo, several spheres were observed by professional divers through a 13 year period, as spheres been bumping into, and getting entangled in, aquaculture nets.

One French diver reports, «despite diving every week at Le Mejean, Frapao, throughout the year in the same area of the Mediterranean, only one sphere has been seen (15 August 2014). There is a strong seasonal thermocline there and the temperature is usually higher than 22 °C at depths of 5–10 m». The one sphere seen there had a brown streak, or structure, in the middle (Pers. com. Edouard Bard).

## Discussion

### Confirmation of species, using DNA analysis

Because the DNA of our sphere samples matches that of adult squid identified as *I. coindetii* from Norwegian waters we infer that the spheres are from *I. coindetii*. Much has been written about taxonomic difficulties in *Illex*. The *COI* tree comprises four clades of *Illex*, one of which clearly pertains to *Illex argentinus* (Castellanos, 1960). There are three other described species: *Illex coindetii*, *Illex illecebrosus* (Lesueur, 1821), and *I. oxygonius* Roper, Lu & Mangold, 1969. We labelled our clades A, B, and C, to indicate their correspondence with the findings of Carlini et al.^32^, and assume that each pertains to one of the described species of *Illex.* Carlini was unable to match species to clades, but Clade A not only contains the adults identified in this project as *I. coindetii*, but also contains specimens from the Mediterranean (DQ373941). Since *I. coindetii* is the only species of *Illex* known from the Mediterranean, this is further confirmation of the identity of Clade A, and thus our spheres, as *Illex coindetii.*

Using citizen science from roughly 200 divers secured observations of 90 spheres, including rare tissue samples of four of them, thus enabling a molecular approach towards the first confirmation of egg masses in situ as those of the broadtail shortfin squid, *Illex coindetii*. *Illex coindetii* was named in honour of Dr. Coindet from Geneva in 1851^37^. It took 180 years from the description of the adult to identification of its egg mass in the wild. To our knowledge no whole egg mass of *Illex* spp. has previously been reported from the wild, except by Adolf Naef, who reported on live ommastrephid embryos and paralarvae from Naples, Italy^2^. The embryos were pulled out of a floating spawn or floating egg mass, or as he describes «Fig. 1 und 2 sind aus einem flottierenden Laich gezogene Larven von Ommatostrephiden». These illustrations were later identified as *Illex coindetii* by Boletzky et al.^26^, studying egg development of *I. coindetii* in the laboratory, claiming «The general characteristics of the embryonic developement observed by us match the figures given by Naef (1923 : plts 9–12) of an unidentified egg mass of a member of the Ommastrephidae (Naef 1921)». However, no drawing of the «laich» was provided.

### Challenges collecting in situ material

Huge gelatinous spheres from squid are difficult to study in situ. They are rarely reported, and hard to sample. We have collected 90 sphere observations from ~ 35 years back (~ 1985 to 2019), from an area stretching from the Mediterranean Sea north to the Norwegian Sea, which gives a good illustration on sphere findings of ~ 2.6 sphere observations per year. In addition, the spheres most likely have a short-life span. Life span of spheres spawned and reared in aquaria (between 40 and 120 cm in diameter) of *Todarodes pacificus* (Steenstrup, 1880) is 5–7 days, with the smallest disintegrating first^38^.

### Sphere shape and size

Gelatinous egg masses of cephalopods vary in size and form among species. Some egg masses are spherical, but there are also examples of oblong structures^39–41^. Sphere size may be up to 4 m in diameter^1,5,42^. Ringvold and Taite (op. cit.) collected information on a total of 27 spheres recorded in European waters varying from 0.3 to 2 m in diameter, as also for the additional spheres from this study. The four spheres in our study, confirmed to belong to *I. coindetii*, measured between 0.5 and 1 m in diameter.

Egg mass of another ommastrephid squid, *Todarodes sagittatus*, has yet to be found in situ. The species is known to be larger than *Illex* species, and egg mass is also most probably larger. The largest spheres recorded in our study measured up to 2 m in diameter, but none of these were sampled for molecular analysis, nor were pictures taken. It is uncertain whether they could belong to other species e.g., *Todaropsis eblanae* (Ball, 1841), *Todarodes sagittatus* or *Ommastrephes* sp..

### Dark streak through core

Almost 60% of the spheres had a dark streak through the center. This feature might be ink, one important characteristic of cephalopods, produced by most cephalopod orders. The ink sac with its ink glands produces black ink containing melanin^43^. During fertilization, sperm are released—as well as possibly some ink. Spheres with or without ink may be a result of spheres beeing at different maturity stages^1^, where spheres with ink are freshly spawned. After a while, when embryos starts developing, the whole sphere, including the streak, will start to disintegrate.

Some of us speculate that one function of the streak through the center might serve as visual mimics e.g. of a large fish in order to scare off predators. Other possible functions discussed are also if the streak/structure can be caused by a sphere strengthening structure which is denser or having a higher optical density than the sourrounding structure. A disadvantage with the streak is that it might reveal the whole transparent sphere in the water, visible to e.g. scuba divers.

### Function of the gelatinous matrix

Observations in captivity^3,44^ showed that species within the genus *Illex* produce gelatinous egg masses while swimming in open water. Gel functions as a buoyancy mechanism that prevents eggs from sinking, and complete density equilibration requires many days under most conditions^44^. Such a buoyancy mechanism keeps pelagically spawned eggs of *Illex* in areas where temperatures are most optimal for embryonic development. Optimal environmental conditions will likely have a positive effect on survival of both hatchlings and paralarvae. Despite consistency in where spawning areas are found, interannual variability has been recorded in the main recruitment areas, which could be related to e.g. mesoscale eddies and/or affecting post-hatching dispersal^45^.

Huge spheres are formed of mucus produced by the nidamental glands, situated inside the mantle cavity of the female^46,47^. When fully developed, hatchlings emit an enzyme which starts to dissolve the mucus. Eggs and embryos from our four spheres were covered in sticky gelatinous mass, except for a few specimens (from Arendal, collected 7 August, and Søgne) laying in the petri dish outside the sticky gel, in the surrounding sea water following the tissue sample, and might have been old enough to start producing such enzymes.

When at hatching*, Illex coindetii* eggs are about 2 mm long^26,48^, in line with other ommastrephids^12^. The longest of our embryos (from Arendal, collected 7 August) measured ~ 2 mm, a developed embryo with long proboscis, mantle about ½ of total body length, as well as chromatophores, large eyes and funnel visible (Fig. 3). It could possibly be a hatchling.

### Abiotic factors and locations

The success and duration of embryonic development is related to water temperature. All observations available to date indicate that successful embryonic development for *I. coindetii* takes ca. 10–14 days at 15 °C; this temperature corresponds to the median temperature value reported for Mediterranean Sea midwater^48^. Boletzky et al.^26^ reports on a temperature minimum above 10 °C. Spheres in the Mediterranean were observed in temperatures ranging between 14 and 24 °C. Watermass temperature for one sphere with recently fertilized eggs (Ålesund sample, embryos stage ~ 3) from Norway was 8 °C. It was also observed north of the existing known distribution range for *I. coindetii*, in the Norwegian Sea, at 43 m depth. Most spheres from Norway were observed from July and August, in water mass 10–14 °C, with maximum temperature at 18 °C.

It is unknown whether some of the observed spheres had drifted to water layers unsuitable for the development of the eggs, and, eventually, would have died due to unfavourable abiotic conditions (e.g. transport outside the optimal temperature- or depth range for that particular species), but most likely they were in an area where they would survive. Higher occurrence of sphere sightings from 2017 to 2019, could be a combination of higher abundance of these squid in the area as well as increased knowledge regarding our Citizen Science Sphere Project, and thereby increased reports of observations.

*Illex coindetii* may be considered as an intermittent spawner with a spawning season extending throughout the year, reaching a peak in July–August^18^.

Our sphere observations from all areas were made from March to October: The earliest sphere which can be documented (to month) in the North Sea to date was observed 27 May (2001), and the last sphere was reported on 20 October (2019), coinciding with a study on adult *Illex condetii* from the North Sea where the spawning season has been suggested to be between spring and autumn^49^. However, our data show a peak of sphere observations from July to September (all areas combined), from July to August in Norway and from August to September in the Mediterranean Sea. The two recordings from Galicia in Spain, and Seiano in Italy, were the earliest recordings of the year, observed 24 March (in 2017 and 2019, respectively). For all areas combined, no observations during wintertime (November to February) have been recorded.

### Embryonic development and consistencies of spheres

We collected tissue mass of four different spheres of *I. coindetii,* and embryos in each sphere were at different developmental stages, ~ 3 to 30, according to Sakai et al.^36^ based on *I. argentinus*. The sphere walls of the four spheres were also of different consistencies (Table 2); from Ålesund sphere with recently fertilized eggs and firm, transparent sphere wall to Søgne and Arendal spheres (the latter collected 7 August) with developed embryos and disintegrating sphere walls. The remains of the Arendal sphere was hanging as a long «scarf» in the water. Experienced divers, who previously had seen a few spherical spheres, recognized this disintegrating sphere.

### Function of spheres

Ommastrephidae fecundity is extremely high, and a single sphere may contain thousands to several hundred thousands of eggs^41,50–52^. The function of the spheres is protection and transport of the offspring by sea currents for paralarval dispersal. Inside these gelatinous structures, the eggs and newly hatched paralarvae are protected from predation by e.g. fish, parasite infection and infestation by crustaceans and protozoans during a first relative short period of their lives^5,51^. Bottom trawlers operate in spawning areas of squids, exposing them to a risk of egg loss, as also for our fisherman at Askøy, Norway, who caught a sphere in his trawl^1,5^.

### Scientific cruises and fishery

The Institute for Marine Research in Norway started identification of cephalopods on their regular scientific cruises in 2013, but no *Illex coindetii* was recorded that year. However, data show increasing catches from 2014 to 2019 (unpublished). No spheres are reported from Norway in 2013, but between 1995 to 2010, and from 2015 to 2019, observations were made. Most observations are between 2017 and 2019, indicating more frequent squid visits/spawnings. This coincides with more frequent sphere observations from 2017 to 2019.

The broadtail shortfin squid, *Illex coindetii,* is probably the most widespread species found on both sides of the Atlantic and throughout the Mediterranean Sea^12^. In the NE Atlantic, it has been reported from Oslofjorden, Norway (59°N);^53^ and the Firth of Forth, east Scotland^54^, southwards along the European and African coasts to Namibia, including Hollam’s Bird Island (24°S) and Cape Frio (18°S)^55^. For example, *I. coindetii* is periodically very abundant in coastal waters of the eastern North Atlantic off Scotland, Ireland and Spain, where it supports opportunistic fisheries. However, the oceanographic and biological factors that drive this phenomenon, are still unknown^12^.

*Illex coindetii* is widely distributed throughout the Mediterranean Sea^11^, where it is caught commercially mostly by Italian trawlers, usually as a by-catch, but also by recreational fishing, by means of squid jigging. Annual Italian landings during the last five years have varied between two and three thousand tonnes, but with historical landings reaching numbers of more than eight thousand tonnes during the 1980s and 1990s (FAO 2019)^15^.

In the North Sea, studies show that inshore squids (*Alloteuthis subulata* (Lamarck, 1798) and *Loligo forbesii* Steenstrup, 1856) are more abundant than short-finned squid (*Illex coindetii, Todaropsis eblanae* and *Todarodes sagittatus*), and *I. coindetii* is among the rarest ommastrephid species caught^49,56^. However, two recent studies (1) on summer spawning stock of *Illex coindetii* in the North Sea^57^ and (2) *I. coindetii* recorded from the brackish Baltic Sea^58^ suggest more frequent visits to this area. Reports on *Illex coindetii* from Norwegian waters are scarce, but it has been reported from Oslofjorden^53^, and recently as by-catch from Stavanger area, and by divers from Oslofjorden and Bergen.

## Conclusion

Without tissue samples from each and every record for DNA corroboration we cannot prove that all spherical egg masses are conspecific, or that the rest of them (86 spheres) also belong to I. *condetii.* However, given similarities between all these spheres and known egg masses spawned by ommastrephid squid, we are confident in attributing these to ommastrephids.

We would like to continue recording sphere observations, hopefully revealing egg masses of other ommastrephids, such as e.g. *Todarodes sagittatus* and *Todaropsis eblanae—*of which egg mass has yet to be described.

## Supplementary Information

Supplementary Video 1.

Supplementary Video 2.

Supplementary Video 3.

Supplementary Video 4.

Supplementary Video 5.

Supplementary Video 6.

Supplementary Captions.

## Data Availability

The datasets generated during and/or analysed during the current study are available from the corresponding author on reasonable request.
