# Supplementary material for: In situ recordings of large gelatinous spheres from NE Atlantic, and the first genetic confirmation of egg mass of Illex coindetii (Vérany, 1839) (Cephalopoda, Mollusca)
Source: Sci Rep. 2021 Mar 30;11:7168. doi: 10.1038/s41598-021-86164-8 (PMC8009939; doi:10.1038/s41598-021-86164-8)
Supplement: Supplementary file 7 — Supplementary Captions. [file 41598_2021_86164_MOESM7_ESM.docx]

Video 1 – Norway, Arendal 7 August (Credit: Anita & Geir Eliassen)

Video 2 – Norway, Arendal 8 August (Credit: Anita & Geir Eliassen)

Video 3 – Norway, Søgne (Credit: Karl Klungland)

Video 4 – Spain, Mallorca, Mediterranean Sea. (Credit: José Coronel)

Video 5 –Spain, Galicia. (Credit: Eduardo Losada Lage)

Video 6 – Norway, Ørstad. (Credit: Ronald Raasch).
